# Composition of the vaginal microbiota during pregnancy in women living in sub-Saharan Africa: a PRISMA-compliant review

**DOI:** 10.1186/s12884-021-04072-1

**Published:** 2021-09-03

**Authors:** Naomi C. A. Juliana, Remco P. H. Peters, Salwan Al-Nasiry, Andries E. Budding, Servaas A. Morré, Elena Ambrosino

**Affiliations:** 1 Department of Genetics and Cell Biology, Faculty of Health, Medicine and Life Sciences, Research School GROW (School for Oncology & Developmental Biology), Institute for Public Health Genomics, Maastricht University, Maastricht, Netherlands; 2 Department of Medical Microbiology, University of Pretoria, Pretoria, South Africa; 3 Department of Medical Microbiology, School for Public Health and Primary Care (CAPRHI), Maastricht University, Maastricht, Netherlands; 4 Research Unit, Foundation for Professional Development, East London, South Africa; 5 Department of Obstetrics and Gynecology, GROW School of Oncology and Developmental Biology, Maastricht University Medical Centre (MUMC), Maastricht, Netherlands; 6 Inbiome, Amsterdam, Netherlands; 7 Laboratory of Immunogenetics, Department Medical Microbiology and Infection Control, Location AMC, Amsterdam UMC, Amsterdam, Netherlands

**Keywords:** Vaginal microbiota, Vaginal microbiome, Vaginal dysbiosis, Pregnancy, Sub-Saharan Africa, Africa

## Abstract

**Background:**

The vaginal microbiota (VMB) are the set of microorganisms residing in the human vagina. During pregnancy, their composition is *Lactobacillus*-dominant in most Caucasian women. Previous studies suggest that the VMB of women with African ancestry is more likely to be non-*Lactobacillus* dominant (dysbiotic) compared to other populations, and possibly relate to the high incidence of pregnancy complications, such as preterm birth. This work reviewed the literature on VMB composition in pregnant women from sub-Saharan Africa.

**Methods:**

A search was conducted in PubMed and Embase databases following PRISMA guidelines. Observational and intervention studies analysing VMB communities from sub-Saharan African pregnant women using molecular techniques were included.

**Results:**

Ten studies performed in seven sub-Saharan African countries were identified. They independently showed that *Lactobacillus*-dominant VMB (particularly *L. iners* or *L. crispatus*) or VMB containing Lactobacilli are the most prevalent, followed by a more diverse anaerobe-dominant VMB, in the studied populations. The majority of pregnant women with a sexually-transmitted infection had a *Lactobacillus*-dominant VMB, but with a significantly higher presence of anaerobic species.

**Conclusion:**

In agreement with studies performed in other populations, *Lactobacillus* species are the most prevalent VMB species during pregnancy in sub-Saharan African women. The frequency of diverse anaerobe-dominant VMB is high in these populations. In Africa, studies on VMB in pregnancy are scant, heterogeneous in methodology, and knowledge remains limited. More insights on VMB composition and their possible sequalae among these populations is needed.

**Supplementary Information:**

The online version contains supplementary material available at 10.1186/s12884-021-04072-1.

## Background

The number of studies on the microbial communities residing in the human vagina, the vaginal microbiota (VMB), and their role in female reproductive health has increased over the past two decades. Recent developments in molecular biology offer innovative opportunities for VMB profiling, but a full understanding of its role in reproductive health is still missing [1]. Usually, the VMB communities are characterized by the most dominant bacterial species, as it is the case for the Community State Types (CSTs) classification [2, 3]. Studies in North America and Europe consistently show that most women of reproductive age, irrespective of the pregnancy status, have a VMB dominated by one of four *Lactobacillus* species: *Lactobacillus iners*, *Lactobacillus crispatus*, *Lactobacillus gasseri*, or *Lactobacillus jensenii* [2, 3]. Lactobacilli can control the vaginal pH levels by producing lactic acid, which is one of the mechanisms maintaining eubiosis and protecting the vaginal milieu from pathogens.

There is increasing evidence that VMB dominated by *L. crispatus*, *L. gasseri*, or *L. jensenii* relate to a healthy vaginal state, and that an overgrowth of (facultative) anaerobes, such as *Gardnerella*, *Atopobium,* and *Prevotella* species contributes to a dysbiotic vaginal state. A dysbiotic vaginal state is often defined as a prolonged deviation from a low-diversity, Lactobacilli-dominated VMB [4]. This vaginal state and its associated bacteria have been linked to the vaginal condition bacterial vaginosis (BV) [5]. While most *Lactobacillus* species are associated with a healthy vaginal state, low proinflammatory cytokine production, and desirable birth outcomes [6, 7], it remains unclear what type of vaginal state is associated with an *L. iners*-dominated VMB [8–10]. *L. iners* is generally considered a vaginal symbiont, but it can also be a potential opportunist pathogen [9]. Furthermore, *L. crispatus* has been shown to hinder colonization by anaerobe bacteria such as *G. vaginalis*, whereas *L. iners* co-exists with such aerobic microorganisms [11]. However, it is not yet clear whether harboring facultative anaerobic bacteria should always be categorized as an unhealthy vaginal microbiota state, as this microbial composition had also been observed in symptoms-free women [11].

Recent studies show that women from African countries or African ancestry more often have a VMB composition that contains BV-related bacteria compared to Caucasian women [2, 3, 12–16]. In these studies, the VMB composition was dominated by *Gardnerella vaginalis, A. vaginae,* and other anaerobic species instead of by *Lactobacillus* species [2, 3, 12–16]. Some of these studies observed that most women from sub-Saharan African or with sub-Saharan African ancestry that displayed a non-*Lactobacillus*-dominant VMB did not have any clinical symptoms associated with bacterial vaginosis. This suggests the possibility of a healthy non-Lactobacilli-dominant VMB state [2, 12, 17, 18]. It is possible that ethnicity, among other factors, influences the VMB composition, through host-genetic factors.

Understanding the role of VMB in health and disease becomes complex when comparing women with different ethnic backgrounds [2, 14, 17, 19]. Besides ethnicity also, hormones influence the VMB. In early pregnancy, changes in hormone levels affect the VMB composition: *Lactobacillus* species are increased and *G. vaginalis* and other (facultative) anaerobic bacteria progressively decrease from the first to the third trimester [17, 19–22]. Following these changes, the VMB remains relatively stable (especially compared to the non-pregnancy state) along most of the pregnancy. During pregnancy changes commonly entail transitions between different Lactobacilli species [15, 21]. However, even in pregnancy, the frequency of *Lactobacillus*-dominant VMB among women of African ancestry is lower compared to women of European ancestry. Furthermore, women of African ancestry are more likely to switch from *Lactobacillus* dominated VMB to a VMB commonly associated with vaginal dysbiosis [15]. Vaginal dysbiosis has been related to various adverse pregnancy outcomes, including preterm birth and higher susceptibility to sexually transmitted infections (STIs) [1, 18, 23–25].

An exhaustive understanding of what constitutes a healthy/normal VMB is lacking. Such knowledge, including appreciation of how VMB composition might impact pregnancy outcomes, is of great importance in areas where the overall burden of pregnancy complications and infections is high [26, 27]. Sub-Saharan Africa suffers a particularly high burden of these conditions: this region has an estimated preterm birth rate of 12% in 2014, accounting for 25% of all preterm births globally. Between 2010 and 2015, the estimated STI prevalence in pregnancy was up to 4.6% for *Neisseria gonorrhoeae* (NG), 6.5% for syphilis, 7.2% for *C. trachomatis* (CT), 25% for *Trichomonas vaginalis* (TV), and mother-to-child transmission of the human immunodeficiency virus (HIV) ranged between 5 and 30% in sub-Saharan Africa [1, 18, 23, 28–31].

There is increasing research interest in understanding how the VMB composition might contribute to, and offer a predictive diagnostic potential for reproductive health outcomes. To date, most VMB pregnancy-related studies and reviews are based on data from North-American and European cohorts of women. Factors influencing the VMB, such as the host (including host-genetics), behavioural (including sexual and cleaning practices), sociodemographic, nutritional, and environmental ones, are nonetheless different across geographical regions and cultures [14, 32, 33]. Comparing VMB composition across women of different populations and extrapolating data from one to explain health outcomes on others might not be an accurate approach [8, 34].

This review aims to compile available data from original research that characterized the VMB composition among pregnant women living in sub-Saharan Africa. Insights from this review are expected to offer background evidence to assist in designing future observational studies to investigate the VMB’s role on pregnancy outcomes and STIs. These are also expected to assist the design of future VMB-related intervention studies that aim to decrease vaginal dysbiosis and its related health problems in sub-Saharan African countries.

## Methods

Searches according to the Preferred Reporting Items for Systematic Review and Meta-Analysis Statement (PRISMA) guidelines were conducted in PubMed/Medline and Embase (Ovid) among studies published up to July 15th, 2020 (Supplementary Table S1) [35]. The used following keywords were used: “vaginal microbiome”, “vaginal microbiota”, “vaginal dysbiosis”, “bacterial vaginosis”, “Africa”, “sub-Saharan Africa”, “pregnant women”, “pregnancy”, and “pregnancy outcome”. To ensure all of the available studies were included, keywords “Africa” or “sub-Saharan Africa” were switched to the individual name of the 48 sub-Saharan African countries (as defined by the World Bank) [36]. The exact Medical Subject Heading (MeSH) and Embase subject heading (Emtree) terms, free-text terms, and combinations of these keywords are compiled in Supplementary Table S1. Lastly, bibliographies of articles with information about BV or VMB were examined, even if articles were excluded, to retrieve potential articles via snowballing. Cohort and intervention studies that reported the VMB composition in women living in sub-Saharan African countries were reviewed. Inclusion criteria were: studies on human participants, pregnancy at the time of sampling, participants recruited in sub-Saharan African countries, as per World Bank classification [36]. Studies were excluded when data and analysis from pregnant and non-pregnant women were combined and when they performed culture-dependent VMB analysis and did not use molecular techniques. Reviews, case reports, abstracts from conferences and case series were also excluded. Studies were not restricted based on specific participant’s characteristics, language or date of publication. The VMB results from molecular techniques reported at phylum, genus, or species level were included in this review. Characterization of vaginal microbial communities based on species’ relative abundance or clustering reported by the studies were also summarized. Summary of VMB composition data from the intervention or case groups and placebo or non-intervention/control groups were reported separately in this review. Data from the non-interventional/control group might be representative of the VMB at the population-level. Data from the case group might inform on women’s VMB status with specific health conditions, such as STIs, or taking compounds that can potentially modulate the VMB, such as medication or supplements.

## Summary and discussion of results

### Study characteristics of vaginal microbiota studies

Searches in PubMed and Embase databases yielded 475 records; an additional 135 records were further identified through snowballing. After the screening procedure, ten articles were included in this review (Supplementary Fig. S1). The characteristics of the retrieved studies are summarized in Table 1. These studies used different approaches to characterize the composition of VMB. However, all consistently clustered *Lactobacillus* species in other groups than anaerobic bacteria, e.g. *Gardnerella* species (Tables 2 and 3 and Fig. 2). Only one study characterized bacterial communities dominated by *Streptococci* and *Staphylococci* species, with a smaller proportion of Lactobacilli [37]. All included studies independently showed that a *Lactobacillus*-dominant VMB or VMB containing Lactobacilli are the most prevalent, followed by a more diverse anaerobe-dominant VMB in pregnant women living in South Africa, Burkina Faso, Rwanda, Kenya, Tanzania, Zambia, and Zimbabwe (Fig. 2). Different study populations were included (Table 1); two studies included results of known HIV-infected and HIV-uninfected women [38, 39]. One study included new cases of CT and TV infections in their case-control study [18], and another study tested the VMB of pregnant sex-workers [34]. One study had a group receiving folic-acid and iron supplements and another group only folic acid supplements [40].

**Table 1:** Study characteristics and VMB diversity findings of included articles that characterized the VMB composition in pregnant women in the sub-Saharan African region

***Author. year*** ***Country***	***Study design***	***Study population***	***Number of pregnant participants***	***Mean or range of Maternal age (years)***	***Gestational age at sampling (weeks)***	***Method /technique used to detect microbiota or vaginal microbiota dysbiosis***
*Frank* et al. *2012* [30] *Burkina Faso*	Case-control (nested in within a prospective cohort MTCT prevention clinical trial of azidothymidine and the microbicide benzalkonium chloride.)	HIV-1 infected pregnant women at 36–38 GA weeks (and their live-born children)	64 women (10 whose babies had a MTCT of HIV and 54 with uninfected babies)	21–27	36–38	16S rRNA pyrosequencing
*Borgdorff* et al. *2015* [36] *Rwanda*	Prospective cohort	Sex-workers	61	24 (19–44)	NR	DNA hybridization microarray of 16S rRNA gene probes
*Gautam* et al. *2015* [8] *Kenya*	Multi-country prospective observational cohort study	Pregnant women < 14 GA weeks	15	NR	NR	16S rDNA phylogenetic microarray
*Jespers* et al. *2015* [9] *Kenya.*	Cross-sectional	Pregnant women < 14 weeks gestation	30	24–26	NR	Quantitative PCR
*Jespers* et al. *2015* [9] *South Africa.*			30	24–26	NR	Quantitative PCR
*Bisanz* et al. *2015* [32] *Tanzania*	Open-label study	Healthy pregnant women between 18 and 40 years and a GA 12–24 weeks	−23 women that that received moringa-supplemented probiotic yogurt. − 24 women without intervention.	24	20	Illumina MiSeq sequencing of the V4 rRNA gene region of the 16S rRNA gene
*Brabin* et al. *2017* [33] *Burkina Faso*	double blind, non-inferiority, RCT. -intervention: folic acid + iron supplements -Control: folic acid supplement	Healthy nulliparous women between 15 and 24 years	- 144 women in folic acid+ iron-arm group CST was determined - 136 women in folic acid group CST was determined	17.1 (both groups)	NR (13–15) (both groups)	Schloss wet-lab MiSeq sequencing of the V4 rRNA gene region of the 16S rRNA gene
*McMillan* et al. *2018* [37] *Rwanda*	RCT, blinded and placebo controlled - intervention: probiotic capsules - control: placebo	Healthy pregnant women between 18 and 55 years and a GA < 36 weeks	−18 women in the placebo arm visit at visit one. - 13 women remained in the placebo arm one month after visit 1.	27.6	22 (8–32)	Illumina MiSeq sequencing of the V6 rRNA gene region of the 16S rRNA gene
*Price* et al. *2019* [35] *Zambia*	Cross-sectional	Pregnant women < 24 GA weeks	256	27 (22–32)	18 (17–19)	Whole genome shotgun sequencing
*Masha* et al. *2019* [18] *Burkina Faso*	nested case-control study - Cases: TV or CT cases - Controls: women that were negative for TV, CT, and bacterial vaginosis	Pregnant women, 18–45 years, ≥14 weeks, and resident of the study area	51 − 18 TV cases − 14 CT cases − 21 control	NR. In category groups: TV cases: - 18-24: 38.9% - ≥ 25: 6.1% CT cases: - 18-24: 64.3% - ≥ 25: 35.74% Control: - 18-24: 28.6% - ≥ 25: 71.4%	NR. In category groups: TV cases: - 14-27: 55.6% - ≥ 28: 44.4% CT cases: - 14-27: 53.9% - ≥ 28: 46.2% Control: - 14-27: 66.7% - ≥ 28: 33.3%	Ion 16S MetagenomicsTM Kit primer set for V2–4-8 amplification of the 16S rRNA gene
*Gudza-Mugabe* et al. *2019* [31] *Zimbabwe*	Cross-sectional study design	Pregnant women > 18 years and a GA of 15–35 weeks	356 (42 HIV-infected and 314 HIV-uninfected women)	29 (24–34)	29 (25–33)	Sequencing of the V4 hypervariable regions of the 16S rRNA gene

16S ribosomal ribonucleic acid (rRNA) gene. *ANC* antenatal care, *ART* Antiretroviral therapy, *BV* bacterial vaginosis, *CI* confidance interval, *CT C. trachomatis*, *GA* gestational age, *HIV* human immunodeficiency virus, *MTCT* mother-to-child transmission, *NR* not reported, *OR* odds ratio, *PCR* Polymerase Chain Reaction, *RCT* randomized control trail, *TV Trichomonas vaginalis*, *VMB* vaginal microbiota, *NR* not reported

**Table 2:** Different types of vaginal microbiota *Lactobacillaceae* clusters in pregnant women in the sub-Saharan African region

*Included articles that used VMB clustering*	*Price* et al.	*Gudza* et al.	*Brabin* et al.	*Borgdorff* et al.	*Gautam* et al.	*Bisanz* et al.	*Frank* et al.	*Donders* et al.
*Name of cluster*	Community state type (CST)			ClusterR	Cluster KRST	Not formulated	Genus-level clustering	morpho-types grades
*Lactobacillus dominant*						✓	Cluster 1	Grade 1
*L.crispatus dominant*	CST-I	CST-I	CST-I	R-I	KRST-I			
*L.gasseri dominant*	CST-II	CST-II						
*L.iners dominant*	CST-III	CST-III	CST-III	R-II	KRST-II			
*L.jensenii*	CST-V	CST-V						

*CST* community state types numeric system described by Ravel et al., [2]. R-clusters, vaginal microbiota cluster method in Rwandese women as described in Borgdorff et al. [36]. KRST-clusters, vaginal microbiota cluster method in Kenyan, Rwandese, South African and Tanzanian women as describe in Gautam et al. [8]

**Table 3:** Different types of vaginal microbiota *diversity* clusters in pregnant women in the sub-Saharan African region

*Included articles that used VMB clustering*	*Price* et al.	*Gudza* et al.	*Brabin* et al.	*Borgdorff* et al.	*Gautam* et al.	*Frank* et al.	*Donders* et al.
*Name of cluster*	Community state type (CST)			ClusterR	Cluster KRST	Genus-level clustering	morpho-types grades
*Diversity group: Higher proportions of strictly anaerobic bacteria, including Prevotella, Dialister, Atopobium, Gardnerella, Megasphaera, Peptoniphilus, Sneathia, Eggerthella, Aerococcus, Finegoldia, and Mobiluncus.*	CST-IV						
*Diversity group: High abundances of Gardnerella, Prevotella and Atopobium species and lower abundances of Dialister, Megasphaera and Mobiluncus species and BVAB1 and the presence of a lower abundance of L. iners.*				R-III	KRST-III		
*Diversity group: Higher proportions of strictly anaerobic bacteria, especially G vaginalis, BVAB1.*		CST-IVA					
*Diversity group: Higher proportions of strictly anaerobic bacteria, especially G vaginalis, L iners, and A vaginae.*		CST-IVB					
*Diversity group: High abundances of Gardnerella, Prevotella and Atopobium species and lower abundances of Dialister, Megasphaera and Mobiluncus species and BVAB1, L. iners, and high abundance Gardnerella genus.*	CST-IV-I			R-IV	KRST-IV		
*Diversity group: High abundances of Gardnerella, Prevotella and Atopobium species and lower abundances of Dialister, Megasphaera and Mobiluncus species and BVAB1, and lower total bacterial abundance than the other mixed anaerobic clusters.*				R-V	KRST-V		
*Diversity group: High abundances of Gardnerella, Prevotella and Atopobium species and lower abundances of Dialister, Megasphaera and Mobiluncus species and BVAB1, L. iners, and highest levels of Prevotella species.*				R-VI	KRST-VI		
*Diversity group: Higher proportions of strictly anaerobic bacteria and G. vaginalis*, *and A. vaginae.*	CST-IV-II						
*Diversity group: Higher proportions of strictly anaerobic bacteria other than G. vaginalis and A. vaginae.*	CST-IV-III						
*Diversity group: pooled dysbiotic clusters.*					KRST-pIII-V		
*Diversity group: Mixed of variety of genera, dominated by Gardnerella spp. and Lactobacillus spp.*						Cluster 3	
*Diversity group: low Lactobacillus spp. and diverse anaerobic groups (eg, Prevotella, Sneathia, Peptostreptococcus)*						Cluster 4	
*Diversity group: lesser Lactobacillus spp mixed with other bacteria.*							Grade II
*Diversity group: Absence of Lactobacillus spp or overwhelming presence of other bacteria (not specified).*			CST IV				Grade III
*Diversity group: Dominated by coagulase-negative staphylococci (S. haemolyticus and S. epidermidis) with lesser quantities of lactobacilli*						Cluster 2	

*CST* community state types numeric system described by Ravel et al., [2]. R-clusters, vaginal microbiota cluster method in Rwandese women as described in Borgdorff et al. [36]. KRST-clusters, vaginal microbiota cluster method in Kenyan, Rwandese, South African and Tanzanian women as describe in Gautam et al. [8]

In comparison, two other studies had given their intervention group probiotic (live organisms intended to have health benefits) capsules, and their control group placebo or no intervention [39, 41]. The rest of the studies did an observational or cross-sectional analysis of one or more populations [16, 17, 42]. Three of the studies used the common VMB classification method by CSTs, as first described by Ravel et al. [2]. Two other studies developed, via the same manner, the Rwanda (R)-cluster and Kenya, Rwanda South Africa, Tanzania (KRST)-clusters [16, 43]. No consistent set of VMB clusters, particularly when it comes to the most diverse VMB, was identified across the included studies (Tables 2 and 3) [2, 16, 38, 40, 42, 43].

### The vaginal microbiota throughout the pregnancy among pregnant African cohorts

Pregnancy is characterized by high circulating estrogen levels produced by the ovaries and later by the placenta [44]. High levels of estradiol promote glycogen deposition in the vaginal epithelium that supports proliferation of various *Lactobacillus* species abundance [3, 45]. Both estrogen levels and microbiota composition change over the course of the pregnancy. Therefore, it is essential to consider the stage of pregnancy at sample collection when interpreting or comparing findings of different studies because VMB dysbiosis at the end of the pregnancy might not have the same impact as VMB dysbiosis earlier on [1, 46, 47]. Previous studies in North America showed that the diversity of VMB is highest during the first trimester of pregnancy and declines during the second and third trimester, possibly due to rising estrogen levels [48–50].

**Fig. 1:**
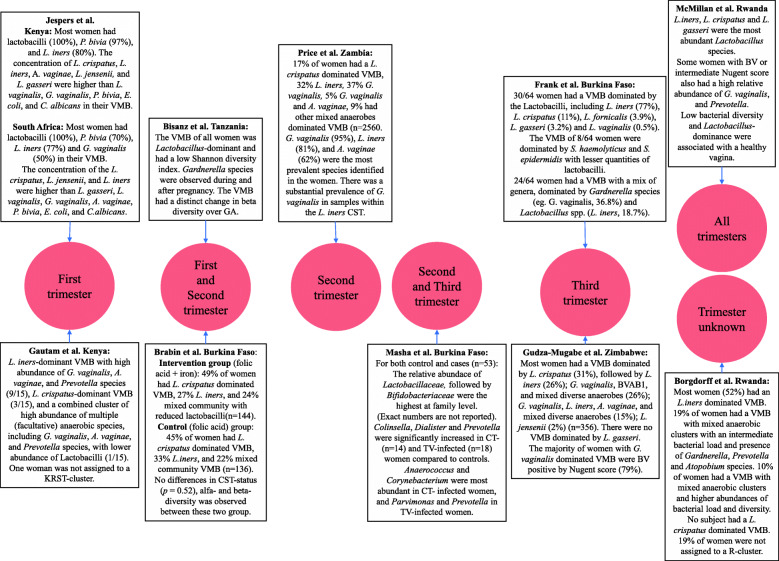
Main findings of vaginal microbiota in studies conducted among sub-Saharan African pregnant women. Illustration is based on collection timepoint (trimesters) during pregnancy. BV, bacterial vaginosis. BVAB1, Bacterial Vaginosis Associated Bacteria 1. CST, community state types numeric system described by Ravel et al. [2]. CT, *C. trachomatis*. KRST-clusters, vaginal microbiota cluster method in Kenyan, Rwandese, South African and Tanzanian women as describe in Gautam et al. [8]. R-clusters, vaginal microbiota cluster method in Rwandese women as described in Borgdorff et al. [38]. TV, *Trichomonas vaginalis*. VMB, vaginal microbiota

Two studies collected specimens in the first trimester (< 13 weeks gestational age (GA)) (Fig. 1). In Kenyan women less than 14 weeks of gestation, the most common VMB cluster observed was *L. iners*-dominant with a high abundance of *G. vaginalis*, *A. vaginae*, and *Prevotella* species (60%); this was followed by an *L. crispatus*-dominant VMB cluster (20%) [16]. Jesper et al. only reported the presence of microorganisms and not their relative abundance, and observed that the *Lactobacillus* genus was present in all 30 Kenyan pregnant women at 14 weeks of gestation, followed by *P. bivia* (97%), *L. iners* (80%), and *G. vaginalis* (50%) [17]. These microorganisms were also observed in 30 South African pregnant women at 14 weeks of gestation. However, in this latter cohort, fewer women (70%) had *P. bivia* in their VMB [17].

Two studies reported the VMB of pregnant women in the first and the second trimester (13–27 weeks GA) (Fig. 1). Bisanz et al. reported that all 42 tested pregnant Tanzanian women around 20 weeks of gestation (range 12–24 weeks GA) had a *Lactobacillus*-dominant VMB [39]. Between 13 and 15 GA weeks’ *Lactobacillaceae* were again observed as the dominant VMB bacterial family among 478 Burkinabe pregnant, with a *Lactobacillus*-dominant VMB present in 78% of participants, *L. crispatus* in 44.9,% and *L. iners* in 33.1% [40].

Moreover 96 HIV-infected and 158 HIV-uninfected Zambian pregnant women had a *Lactobacillus*-dominant VMB, either by *L. iners* (32%) or *L. crispatus* (17%) during the second trimester of the pregnancy [42] (Fig. 1). Ninety-five percent of women carried *G. vaginalis*, and 42% of women had a *G. vaginalis*-dominant VMB [42].

Masha et al. included samples collected in the second and third trimester (≥14 weeks GA) and identified *Lactobacillaceae* as the dominant VMB bacterial family among 53 pregnant Kenyan women [25] (Fig. 1).

The two studies that reported the VMB composition at the third pregnancy trimester (> 27 weeks GA) also reported that most Burkinabe and Zimbabwean women had *Lactobacillus*-dominant VMB. In Burkina Faso, 47% of women had a *Lactobacillus*-dominant VMB, while 37.5% of them had a VMB dominated by both *Gardnerella* species and *L. iners* [37]. In 314 HIV-uninfected and 42 HIV-infected Zimbabwe pregnant women, the *Lactobacillus* species were further identified. The majority of pregnant women in that cohort had a VMB dominated by *L. crispatus* (31%), followed by a diverse VMB with various mixed anaerobes bacteria, including *G. vaginalis* (41%) [38]. Another study identified *Lactobacillaceae* as the dominant VMB bacterial family among 38 Rwandan pregnant women across all trimesters (20 women who received *Lactobacillus rhamnosus* GR-1 and *Lactobacillus reuteri* RC-14 and 18 women who received placebo) [41] (Fig. 1).

Lastly, Borgdorff et al. did not report sampling time [43] (Fig. 1). But like the other studies, they observed that during pregnancy *Lactobacillus* species were most prevalent among 21 Rwandese pregnant women, in particular *L. iners* [43]. The second most prevalent VMB composition in Rwandese pregnant women were women with diverse anaerobic communities and BV-related bacteria, such as *Gardnerella* and *Prevotella* species [41, 43].

These sub-Saharan African studies, irrespective of sampling time and population reported that most women had a VMB *Lactobacillus*-dominant VMB composition or high concentrations of *Lactobacillus* species in their VMB. However, based on the higher frequency of a diverse anaerobic VMB in the sub-Saharan African population and women with African ancestry, two hypotheses were drawn [13, 15, 51]. Firstly, it might be that the role of hormones, in particular estradiol, is not as influential on the VMB during pregnancy as host-genetic factors. Secondly, since a diverse anaerobic VMB is common among non-pregnant sub-Saharan African women [2, 3, 12–16], the VMB composition during the pregnancy might remain diverse because there is already a high presence of anaerobic bacteria before pregnancy. Future longitudinal studies containing information on the VMB composition before, during, and after pregnancy might provide information on the VMB composition changes between these periods and whether the VMB before pregnancy influences the VMB during pregnancy in sub-Saharan African populations.

### Vaginal microbiota communities by clustering

**Fig. 2:**
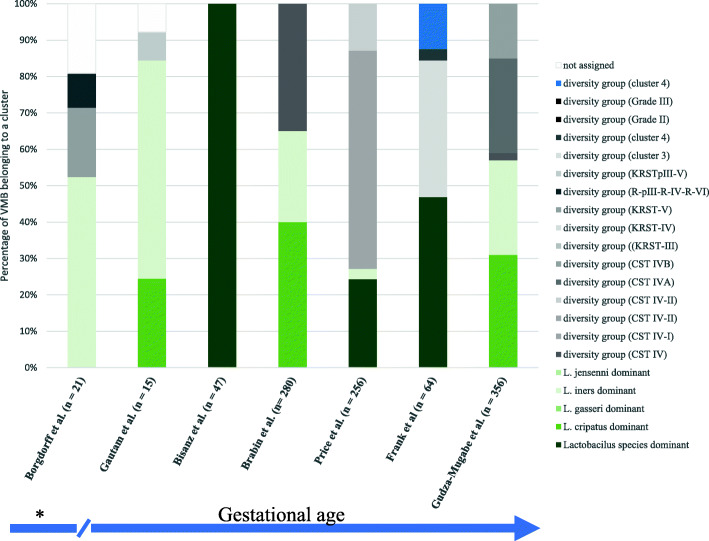
Percentages of vaginal microbiota clusters from pregnant women living in sub-Saharan Africa. *Unknown time of exact sampling. Bacterial names and clusters abbreviations are corresponding to data in Table 2

To date, there is no consensus on how best to classify VMB communities [46] (Tables 2 and 3). Most of the studies in our review showed that a *Lactobacillus*-dominant VMB is the most prevalent vaginal microbiota in tested pregnant women despite differences in methodology and definitions [16, 25, 37–43, 52]. This evidence is in line with findings from other populations in Asia, North-America, and Europe [3, 19, 53]. Independent of the type of clusters used to classify the VMB and the gestational age at sampling when speciated, *L. iners* was the most pervasive *Lactobacillus* species detected, followed by *L. crispatus* [16, 17, 37, 39–42] (Fig. 2). Unlike pregnant cohorts in Europe and North America, the second most prevalent VMB composition detected in Burkina Faso, Rwanda, and Zambia were diverse VMB clusters with high abundances of anaerobic bacteria, in most cases *G. vaginalis* [3, 19, 37, 42, 43]. Several factors have been proposed to contribute to women becoming tolerant to a non-*Lactobacillus* dominant vaginal milieu, such as genetic variation, like polymorphism in the immune and hormone-response genes, for instance single nucleotide polymorphisms in toll-like receptor (TLR)-4 (rs1554973 and rs7856729 or anti-inflammatory interleukin-10 (*IL10*–819 T/T and *IL10*–1082 A/A) [54–59]. Several studies observed that host genetics, via polymorphism in immune-related genes, increases colonization of specific bacteria in the vagina [60–63]. Interestingly, women with African ancestry carry polymorphisms in cytokine genes, making them more susceptible to inadequately respond to lipopolysaccharides (LPS) and develop vaginal dysbiotic conditions, such as BV [24, 64]. The high frequency of a more diverse VMB and anaerobic composition observed among sub-Saharan African (pregnant) women are in agreement with the high prevalence of BV (proximally 40%) also observed among sub-Saharan African women [14, 33, 55]. Early-life exposure to non*-Lactobacillus* dominant VMB may mediate the host immunological reaction to microbiota species and nurture immunological tolerance [55]. Genetic variations and host immunological mediators may partially clarify the VMB composition variability across ethnicities, but more evidence is warranted [3, 13, 64].

In pregnant Zimbabwean women, the prevalence of *L. crispatus*-dominant VMB was slightly higher than the prevalence of *L. iners*-dominant VMB or VMB with a high abundance of *G. vaginalis,* Bacterial vaginosis-associated bacterium *1* (BVAB1), and mixed diverse anaerobic bacteria [38]. Interestingly, none of the pregnant Rwandan sex-workers had an *L. crispatus* dominant VMB [43]. These observations are in line with results from pregnant African-American women in which *L. iners* and diverse anaerobic VMB were the most dominant VMB [13, 51]. Unfortunately, Jesper et al., Bisanz et al., and Masha et al. did not report the dominant species in the VMB of their South-African, Kenyan, Tanzanian, or Burkinabe pregnant cohort [17, 25, 39]. Masha et al. and Bisanz et al. independently characterized the VMB based on the genus and reported separately that the relative abundance of the *Lactobacillus* genus was the highest in their cohort from Burkina Faso and Tanzania, respectively [25, 39]. Their findings are in concordance with the results of Brabin et al. also reporting a high prevalence of *Lactobacillus*-dominant VMB clusters (*L. crispatus* and *L. iners*) in Burkinabe women [40]. Jesper et al. did report that all pregnant women tested in both countries had *Lactobacillus* genus in their VMB, *P. bivia* (70%), and *G. vaginalis* (50%) were also detected in the majority of women in South Africa [17]. In Kenya, 97% of pregnant women had *P. bivia* present in their VMB [17]. Petrova et al. reviewed and proposed how the various VMB composition clusters, as classified by CSTs, can be associated with vaginal health or dysbiosis [9]. It has been suggested multiple times that VMB dominated by *L. crispatus*, *L. gasseri*, or *L. jensenii* relates to a healthy vaginal state and an overgrowth of anaerobes as *Gardnerella*, *Atopobium,* and *Prevotella* species most likely contributes to a dysbiotic-BV state. However, it is unclear whether the VMB clusters consistent with the presence of modest *Lactobacillus* species with a higher relative abundance of anaerobic bacteria, such as BVAB1, *G. vaginalis*, *A. vaginae*, but without an overgrowth of anaerobes bacteria, associate with a healthy vaginal state or with a transitional state of the vaginal milieu to a BV state [9]. Shifts from eubiosis to dysbiosis, and vice-versa, remain unpredictable processes, and their causes are not yet understood [65].

Moreover, it is unclear what type of vaginal state is associated with a *L. iners*-dominated VMB [8]. Unlike the other *Lactobacillus* species, *L. iners* does secrete some amounts of hydrogen peroxide (H_2_O_2_), but not D-lactic acid, both common secretory products of most Lactobacilli [10]. *L. iners* also been shown to promote the growth of *E. coli* and *G. vaginalis* biofilm and can produce pore-forming toxin similar to *G. vaginalis* can induce lysing of erythrocytes [66–69]. Several data showed that VMB-dominated by *L. iners* are more likely to transition to a BV-associated VMB and offer limited protection against vaginal dysbiosis, clearance of urogenital pathogens, and might be a risk factor to adverse pregnancy outcomes [10, 70, 71]. For example in a cohort from London, *Lactobacillus iners* dominance at 16 weeks of gestation was significantly associated with both a short cervix< 25 mm and preterm birth < 34 + 0 weeks [72]. However, the debate about what composition defines a healthy and unhealthy VMB is ongoing, especially across different ethnic populations. Thus, further investigation should determine what the exact role of an *L. iners*-dominant VMB and a diverse VMB are on pregnancy outcomes, especially in the sub-Saharan population where the prevalence of these VMB compositions are high.

### The VMB composition of women carrying a sexually transmitted pathogen

Globally, 70% of all people living with HIV live in sub-Saharan Africa [24, 55, 73]. The articles by Frank et al. and Price et al. provide an essential insight into the role of HIV in the VMB of pregnant women living in the region with the global highest burden of HIV and other STIs [37, 42]. A high frequency of vaginal dysbiosis were observed in both studies (Fig. 2). In non-pregnancy studies it was reported that the most common clinical diagnosis of BV was associated with a 60% increase in the risk of acquiring HIV after exposed, and that presence of *L. crispatus* associates with suppression of viral replication and virus inactivation [74–77]. As mentioned before, *Lactobacillus* species, other than *L. iners,* are important to vaginal health as they produce antimicrobial molecules. For instance, H_2_O_2_ and other bacteriocins can destroy urogenital pathogens, and lactic acid can inhibit pathogenic bacteria’s growth and disrupt the bacterial cell membranes, thereby contributing to the host immune response to bacterial liposaccharides [74]. Lactic acid is thought to be the main regulator of “healthy” vaginal function, rather than *Lactobacillus* species, since the vaginal tracts of asymptomatic women with a diverse VMB are also dominated by taxa that produce lactic acid [9]. Women with low production of lactic acid and vaginal dysbiosis have a higher risk of acquiring STI when exposed and of adverse reproductive and obstetric sequelae [74, 78].

Luckily, antiretroviral therapy (ART) and associated approaches for the prevention of Mother-To-Child Transmission of HIV infection (MTCT) are becoming more standard practice in most sub-Saharan African settings [79–82]. Price et al. observed that the prevalence of anaerobes dominated VMB, or CST IV, was higher among HIV-infected women taking ART prior to conception (63.5%) and among the non-ART group (85.3%), compared to HIV uninfected participants (45.3%) [42]. They also reported an association between an anaerobic- dominant VMB, maternal HIV infection, and ART timing before or during the pregnancy [42]. Future studies are needed to investigate whether specific interventions, such as ART, antibiotics, and probiotics, can modulate the VMB into a healthy state, which might be different based on ethnicity, and might lower the risk of HIV acquisition and MTCT.

Besides HIV, other STIs are highly endemic in sub-Saharan Africa, including TV and CT, and have been associated multiple times with vaginal dysbiosis, lower Lactobacilli levels, and alkaline vaginal milieu [74, 83–85]. Brabin et al. reported in their study that TV and BV were associated (*P* < 0.01) with a more diverse and anaerobic VMB composition in Burkinabe pregnant women [40].

The included study by Masha et al. characterized the VMB profiles in TV- and CT-infected, and non-infected pregnant women in Kenya [25]. Lactobacilli were the most abundant in all three groups. In CT-infected pregnant women, *Anaerococcus* and *Corynebacterium* were most abundant compared to TV-infected women or controls. In line with other studies’ observations, in TV-infected women, *Parvimonas* and *Prevotella* were most abundant and significantly more present compared to controls [86]. The anaerobes *Prevotella, Anaerococcus, Parvimonas* (formerly *Peptostreptococcus*), and *Dialister* have been observed frequently in women with vaginal dysbiosis (with CST IV) or BV; however, it is still unclear what the role of *Parvimonas*, *Anaerococcus, Dialister*, and *Corynebacterium* is in the pathology of TV and CT infections and its effect on the VMB state [1, 25]. Therefore, the interplay between VMB and STIs should be further investigated in in-vitro models or samples from women living in a high-risk population. Such findings are crucial for vulnerable populations with high STI prevalence, such as pregnant women in sub-Saharan Africa countries.

### Effect of probiotics, vitamins, and mineral on the VMB during pregnancy

Besides the high burden of STIs, malnutrition, or undernourishment is also prevalent in low-income areas in sub-Saharan Africa, especially among pregnant women [87]. Currently, vitamins and minerals (especially folic acid and iron) are recommended during pregnancy for every woman across sub-Saharan Africa, particularly for undernourished women. Brabin et al. did not observe any association between VMB composition, extra iron supplements, and host iron status in pregnant Burkinabe women [40].

McMillan et al. and Bisanz et al. examined whether probiotics containing specific Lactobacilli would alter the VMB composition during pregnancy in and Tanzanian women, respectively [39, 41]. Unfortunately, VMB data solely from the control group was not reported in Bisanz et al. [39]. However, their data were still included in this review because, similar to McMillan et al., they explicitly mentioned that there was no difference in the VMB composition between the probiotic group and the control group [39, 41]. Interestingly, McMillan et al. did report that the Rwandese women in the calcium carbonate placebo group were significantly more likely to give birth preterm than women in their probiotic group [41]. However, both groups’ sample sizes were too small to draw a final conclusion [41]. The relationship between these pregnancy outcomes with their respective VMB was not further analyzed or discussed [41].

Different *Lactobacillus*-based probiotics, made with mixtures of *Lactobacillus* species, *Bifidobacterium,* and *Streptococcus* strains have been studied [23, 88]. Lately, the use of probiotic (containing *Lactobacillus* strains, mostly *Lactobacillus rhamnosus*) have been claimed to restore *Lactobacillus-*dominated VMB in women with BV [89–91]. The two included intervention studies also proved that probiotic supplements are safe for use. However the probiotic adherence (important feature of probiotics associated with their potential for colonization) to the human vagina mucus and the cost-effective strategies for its implementation should also be considered [39, 41]. Ideally, there is need for a cost-effective treatment that targets specific pathobionts (commensal microorganisms with pathogenic potential) or dysbiosis-associated anaerobes while sparing *Lactobacillus* species could restore VMB eubiosis and simultaneously nourish malnourished (pregnant) women in resources-constrained settings.

### VMB characteristics and pregnancy outcome

VMB characteristics, or presence of pathobionts, have been linked to various maternal, fetal health issues and adverse pregnancy outcomes, primarily preterm birth, but also chorioamnionitis, premature rupture of membranes, stillbirth, preeclampsia [21, 92–96]. The retrieved studies that analysed the VMB by molecular approaches did not investigate the association between VMB and an adverse pregnancy outcome. To our knowledge, only one culture-based study analysed the relationship between VMB and late pregnancy complications (after 20 weeks’ gestation) in sub-Saharan African pregnant women, to date. Donders et al. observed that South African women with little or no Lactobacilli were 3.6 times more likely to have an infant with low birthweight (< 2 kg) compared to women with a high abundance of Lactobacilli [97]. Discussion on the mechanisms behind this possible causal effect of VMB dysbiosis on several adverse pregnancy outcomes were previously reviewed and are outside the scope of this review [1, 98]. Nevertheless, there is a need for future studies using newer molecular diagnostic techniques to confirm these findings and determine whether low birthweight, preterm birth, or other adverse pregnancy outcomes are generally associated with maternal VMB composition in sub-Saharan African women.

### Limitations and future considerations

The heterogeneity of results included in this review is high since different study designs were included, different study populations (from sex-workers to people with low sexual and reproductive risk behavior), and studies that used diverse molecular approaches. Pyrosequencing and Illumina MiSeq sequencing of the 16S ribosomal (r) RNA gene allow for an unprecedented high-resolution detection of the microbiota [99]. However, microarray approaches, like that used by Borgdorff et al., cannot detect genes of species that are not a priori included in the array [43]. Furthermore, comparing 16S rRNA gene sequencing results from different studies should also be done carefully since there is a lack of standardization in methodologies used to prepare samples and analyze the VMB results [100]. Also, each variable (V)16S rRNA region has a different resolution to identify bacterial strains; for instance, the V2-V3 fragment has the highest resolution to detect species and genera [101]. These technical differences can also result in under- or overrepresentation of a bacterial taxon [46, 102].

The present review identified many research gaps. It is still unclear what the mechanism behind a healthy and balanced VMB is [46], as it is the role of common vaginal bacteria in pregnant sub-Saharan African women. Furthermore, the number of different dysbiotic clusters reported in this review (Table 2) and the high prevalence of *L. iners* and *G. vaginalis* among pregnant sub-Saharan women might indicate eubiosis or healthy VMB, rather than a transitional or abnormal VMB as seen in other ethnic populations [42, 103]. A multi-country study and a more tailored sub-classification of the VMB characterized by high diversity might be more relevant in the African population than in other populations. The clinical and biological relevance of defining the VMB by clusters also remains to be further investigated in women from sub-Saharan Africa [46]. Deciphering the relationship between host, VMB, and the immune system can even provide therapeutic intervention strategies, for instance, pro- or antibiotics, that might benefit maternal health [1]. To do so in a more personalized manner, the host ethnicity’s role should also be taken into account.

Furthermore, the role of pathogens on VMB composition, especially STIs that have a high incidence and prevalence, the latest estimate was made in 2012, where in Africa there were 357 million new episodes of four curable STIs (chlamydia, gonorrhoea, syphilis, and trichomoniasis) [104]. These STIs also cause a high burden of disease in African communities, should also be further evaluated [24, 25, 38, 42]. Moreover, interventions with dietary strategies, including human colostrum/milk or prebiotics/probiotics, have already been studied in preterm infant and early pregnancy development after in-vitro fertilization [23, 88]. Considering the possible association between individual VMB states and adverse pregnancy outcomes, affordable and easily accessible interventions that beneficially modulate the VMB should be evaluated and are urgently needed [21].

## Conclusion

This study provides an overview of the current knowledge of VMB composition in pregnant women living in seven sub-Saharan Africa. It remains challenging to compare VMB characteristics across studies performed in this region since populations and experimental methods vary considerably [24]. Nevertheless, the evidence provided here highlights that most sub-Saharan African studies reported pregnant women having VMB dominated by *L. iners* or more diverse anaerobic communities, mostly with a high abundance of *G. vaginalis*. Future research should investigate the pathogenesis and host-immunological role of the VMB bacteria on various health conditions and outcomes in more detail. To allow for analysis across studies, consensus on how to test and report VMB composition in sub-Saharan African women should be reached for a better comparison of data sets. Furthermore, a large shared multi-county/international database could also help to minimize these problems. A systematic comparison of evidence across countries will help provide substantial qualitative evidence for public health strategies to improve reproductive and maternal health in sub-Saharan Africa.

## Supplementary Information

**Additional file 1: Figure S1.** PRISMA based Flow diagram displaying the study selection [28].

**Additional file 2: Table S1.** Search strategies and hits based on searches last conducted on July 15, 2020 [29].

## Data Availability

Not applicable.

## Abbreviations

VMB: Vaginal microbiota
CST: Community State Types
BV: Bacterial vaginosis
STIs: Sexually transmitted infections
NG: *Neisseria gonorrhoeae*
CT: *C. trachomatis*
TV: *Trichomonas vaginalis*
HIV: Human immunodeficiency virus
PRISMA: Preferred Reporting Items for Systematic Review and Meta-Analysis Statement
MeSH: Medical Subject Heading
Emtree: Embase subject heading
R-cluster: Rwanda cluster
KRST-cluster: Kenya, Rwanda South Africa, Tanzania cluster
GA: Gestational age
TLR: Toll-like receptor
IL: Interleukin
LPS: Lipopolysaccharides
BVAB1: Bacterial vaginosis-associated bacterium *1*
ART: Antiretroviral therapy
