# Supplementary material for: Composition of the vaginal microbiota during pregnancy in women living in sub-Saharan Africa: a PRISMA-compliant review
Source: BMC Pregnancy Childbirth. 2021 Sep 3;21:596. doi: 10.1186/s12884-021-04072-1 (PMC8418042; doi:10.1186/s12884-021-04072-1)
Supplement: Supplementary file 1 — Additional file 1: Figure S1. PRISMA based Flow diagram displaying the study selection [28]. [file 12884_2021_4072_MOESM1_ESM.docx]

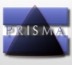
ADDITIONAL FILE 1

**PRISMA 2009 Flow Diagram**

## Eligibility

## Included

## Identification

## Screening

Additional records identified by snowballing sampling (references) (n=135)

Records identified through a search conducted per each individual African country

(n = 228)

Records identified through PubMed (n = 97),

Embase (OVID) (n = 150)

Records excluded based on title and abstract

(n = 567)

- Study not about VMB
- Cohort not from sub-Saharan Africa region
- Participants not pregnant

Records screened
(n = 610)

Full-text articles excluded, with reasons (n = 24)

- Specific chosen group of microorganisms not VMB (n =8)
- Vaginal swab was collected post-delivery (n =4)
- Article with no separate data for pregnant group (n =2)
- Molecular markers/metabolomics (n = 3)
- Other study-design (review/abstract) (n= 2)
- Data included in other article selected (n= 2)
- Not currently pregnancy (n=2)
- Protocol for VMB paper (n=2)
- Cultured based methodology (n= 2)

Full-text articles assessed for eligibility
(n = 43)

Selected articles that are duplicates (n =6)

Studies included in qualitative synthesis
(n = 10)

**Figure S.1.** PRISMA based Flow diagram displaying the study selection [29].
