# Supplementary material for: Composition of the vaginal microbiota during pregnancy in women living in sub-Saharan Africa: a PRISMA-compliant review
Source: BMC Pregnancy Childbirth. 2021 Sep 3;21:596. doi: 10.1186/s12884-021-04072-1 (PMC8418042; doi:10.1186/s12884-021-04072-1)
Supplement: Supplementary file 2 — Additional file 2: Table S1. Search strategies and hits based on searches last conducted on July 15, 2020 [29]. [file 12884_2021_4072_MOESM2_ESM.docx]

ADDITIONAL FILE 2

**Table S.1.** Search strategies and hits based on searches last conducted on July 15, 2020.

| **Database** | **Search strategy** | **Number hits** |
| --- | --- | --- |
| Pubmed | ((((((((Vaginal microbiota[MeSH Terms]) OR vaginal microbiome[MeSH Terms]) OR vaginal microbiota) OR vaginal microbiome) OR vaginal dysbiosis') OR bacterial vaginosis OR aerobic vaginitis)) AND ((africa[MeSH Terms]) OR sub-saharan africa[MeSH Terms])) AND ((((((pregnant women[MeSH Terms]) OR pregnancy[MeSH Terms]) OR pregnant) OR pregnancy) OR pregnancy outcome[MeSH Terms]) OR pregnancy outcome) | 97 |
| Pubmed | (((((((Vaginal microbiota[MeSH Terms]) OR vaginal microbiome[MeSH Terms]) OR vaginal microbiota) OR vaginal microbiome) OR vaginal dysbiosis') OR bacterial vaginosis)) AND ((((("xxx"[Mesh]) OR (“xxx”) OR (“xxx” women)) AND (((((pregnant women[MeSH Terms]) OR pregnancy[MeSH Terms]) OR pregnant) OR pregnancy) OR (((pregnancy outcome[MeSH Terms]) OR pregnancy outcome)))))) | 228 |
| Embase (Ovid) | (exp Vaginal microbiota/ or exp vaginal microbiome/ or exp vaginal flora/ or (vaginal microbiota or vaginal microbiome or vaginal dysbiosis or bacterial vaginosis or aerobic vaginitis).ti,ab,kw.) AND (exp africa/ or exp Africa south of Sahara/ or sub-Saharan Africa.ti,ab,kw.) AND (exp pregnant women/ or exp pregnancy/ or exp pregnancy outcome/ or (pregnant or pregnancy or pregnancy outcome).ti,ab,kw.) | 150 |

At the “xxx” each Sub-Saharan African country was filled in per search, countries selected based on the world bank organization database: Angola, Benin, Botswana, Burkina Faso, Burundi, Cabo Verde, Cameroon, Central African Republic, Chad, Comoros, Congo, Cote D’Ivoire, Equatorial guinea, Eritrea, Eswatini, Ethiopia, Gabon, Gambia, Ghana, Guinea, Guinea-Bissau, Kenya, Lesotho, Liberia, Madagascar, Malawi, Mali, Mauritania, Mauritius, Mozambique, Namibia, Niger, Nigeria, Rwanda, Sao Tome and Principe, Senegal, Seychelles, Sierra Leone, Somalia, South Africa, South sudan, Sudan, Tanzania, Togo, Uganda, Zambia, Zimbabwe [30].
